# How does interprofessional education influence students’ perceptions of collaboration in the clinical setting? A qualitative study

**DOI:** 10.1186/s12909-022-03372-0

**Published:** 2022-04-27

**Authors:** Carolyn Teuwen, Stéphanie van der Burgt, Rashmi Kusurkar, Hermien Schreurs, Hester Daelmans, Saskia Peerdeman

**Affiliations:** 1 Northwest Academy, Northwest Clinics Alkmaar, Alkmaar, the Netherlands; 2 Research in Education, Amsterdam UMC location Vrije Universiteit Amsterdam, Boelelaan 1118, Amsterdam, the Netherlands; 3 Teaching & Learning Centre (TLC) FdG - UvA, Amsterdam UMC location AMC, Amsterdam, the Netherlands; 4 LEARN! Research Institute for Learning and Education, Faculty of Psychology and Education, Vrije Universiteit Amsterdam, Amsterdam, the Netherlands; 5 Department of Surgery, Northwest Clinics Alkmaar, Alkmaar, the Netherlands; 6 Clinical Skills training department, Faculty of Medicine Vrije Universiteit Amsterdam, Amsterdam, the Netherlands; 7 Department of Neurosurgery, University of Amsterdam, Amsterdam UMC location Vrije Universiteit Amsterdam, Amsterdam, the Netherlands

**Keywords:** Interprofessional education, Interprofessional collaboration, Social capital theory, Qualitative method, Perceived influence, Interviews

## Abstract

**Background:**

Interprofessional education (IPE) aims to improve students’ collaborative competencies and behaviour. The effect of classroom IPE on students’ perceptions of collaboration in clinical practice, and how knowledge is possibly transferred, has yet to be investigated. The research question of this study was: How does IPE in a classroom setting influence students’ perceptions of collaboration in clinical practice? Social capital theory is used as the theoretical lens. Social capital theory describes how social relationships generate benefits for the individuals involved. Social capital can be divided into three forms of social cohesion: bonding, bridging and linking social capital. Bonding refers to connections that are close and strong, such as family. Bridging social capital occurs in more distant relationships. Linking social capital refers to relationships between individuals with different power or social status.

**Methods:**

A qualitative study with semi-structured face-to-face interviews was conducted to explore students’ perceptions and experiences. Nursing and medical students who had participated in four classroom IPE-sessions were asked about the perceived influence of the IPE-sessions they had attended on their interprofessional collaboration. Thematic analysis was conducted, with sensitising concepts of ‘bonding’, ‘bridging’ and ‘linking social capital’ from the social capital theory.

**Results:**

Twenty-two interviews were conducted. Students experienced: 1) exchange of discipline specific knowledge, 2) general knowledge about each other’s responsibilities, 3) reduction of hierarchy, and 4) improvement in patient care. The first two themes reflect bridging social capital, since students experience that the other student is from a different group. The third theme reflects linking social capital, since students experience a difference in (social) status. The fourth theme most explicitly reflects ‘getting ahead’ or doing better, what is referred to as an effect of increased social capital.

**Conclusion:**

This study reveals new insights regarding how increased social capital of undergraduate students after IPE-sessions in a classroom setting influences the way they conceptualise and experience interprofessional collaboration in clinical practice. These insights contribute to the understanding of the effectiveness of IPE in undergraduate curricula. Further research on long-term effects is underway.

## Introduction

Interprofessional education (IPE) aims to improve students’ collaborative competencies and behaviour, and thereby prepare students for the care of complex patients [1, 2]. The World Health Organization (WHO) emphasizes the importance of IPE and interprofessional collaboration (IPC) and formulates the following definitions [3]:

- ‘Interprofessional education occurs when two or more professions learn about, from and with each other to enable effective collaboration and improve health outcomes’.
- ‘Collaborative practice in healthcare occurs when multiple health workers from different professional backgrounds provide comprehensive services by working with patients, their families, care givers and communities to deliver the highest quality of care across settings’.

According to the model of interprofessionality by D’Amour & Oandasan [4], IPE can improve patient care by influencing IPC. Most IPE-studies investigate the effect of interventions on IPC with quantitative measures [5–7]. But *how* is this change in IPC, which is a behavioural change, established by an IPE intervention? We know from Beck’s cognitive model that perceptions and ideas influence behaviour [8]. Therefore, a behavioural change of students in clinical practice might be the result of a change in students’ perceptions and ideas. A qualitative study to investigate the effect of IPE on students’ perceptions would add to the literature and understanding of how IPE is effective, and can therefore help to shape powerful IPE-interventions.

Accordingly, the primary research question of this study was: How does IPE in a classroom setting influence students’ perceptions of collaboration in clinical practice?

It is important to determine in which forms IPE should be offered, so that it indeed improves students’ perceptions about collaboration. Several studies suggest IPE is most effective when offered in authentic settings (i.e. clinical practice) [9]. What is not known is whether IPE can also change students’ perceptions when offered in a classroom setting. This might be less authentic, but can be established more easily. Evidence for classroom IPE changing students’ perceptions about collaboration in clinical practice can be helpful in making IPE a substantial component of health professions’ education curricula.

### Social capital theory

Viewing the influence of an IPE-intervention through a theoretical lens can help to understand *why* IPE is effective. One of the theories used to study the ‘how’ of IPE is the theory of social capital [10–12]. Social capital theory describes how social relationships and social networks generate benefits, i.e. resources of knowledge or support, for the individuals involved in them [13]. These social networks are a key component in IPE and IPC [14, 15], since students learn ‘with, from and about’ each other. In other fields social capital is associated with several advantages such as innovation and improved health outcomes [12, 16]. In IPE-research social capital has been used in several case studies [11, 12, 17]. Research about the transfer of knowledge and experiences from IPE *to IPC* is needed, and maybe social capital facilitate this transfer.

**Fig. 1:**
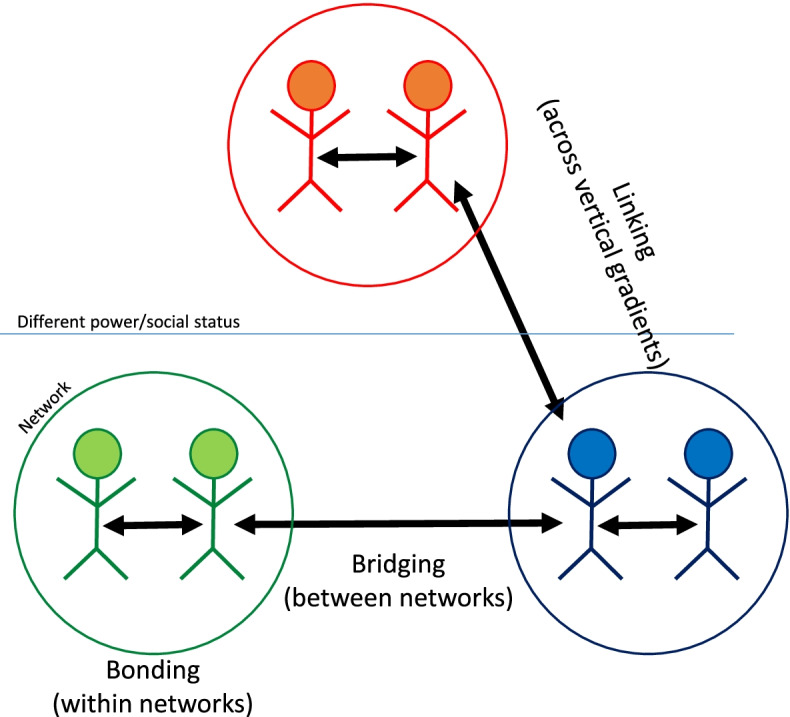
Three forms of social capital (adapted from Aldrich (2012) [18])

Based on levels of social cohesion, social capital can be divided into three forms: bonding, bridging and linking social capital [13]. Bonding refers to connections between people that are close and strong, such as family, close friends and neighbors. It often involves people with the same characteristics and background. Bridging social capital occurs in more distant relationships, with people who are more ‘unalike’, for example with colleagues. Linking social capital refers to ties among individuals that are not only unalike, but also have different power and social status, for example between employers and employees [13]. Figure 1 depicts all these forms of social capital.

In health care, one profession could represent the network circle in Fig. 1 on the left and another profession the network circle on the right. Accordingly, with IPE, an increase of bridging social capital could occur. Van Oorschot et al. (2006) [19] outline that more bridging social capital could narrow the gap between different communities, such as nurses and doctors, and that it can facilitate ‘getting ahead’. In medical practice ‘getting ahead’ can involve better medical professionals and better patient care, but also the access to and acquisition of knowledge that belongs to a different profession than your own. Evaluating IPE and its effectiveness through the lens of social capital can therefore provide valuable insights regarding how interprofessional relationships between students may affect perceptions of collaborative practice.

## Methods

To explore the influence of IPE on students’ perceptions and experiences in clinical practice, we conducted a qualitative interview study. Using a constructivist paradigm, the data were collected/produced and analyzed based on experiences of participants and through interaction between participants and researchers [20].

### IPE-setting

The Northwest Academy is the educational institute of the Northwest Clinics in Alkmaar, the Netherlands. Nursing students that followed their educational program at the Northwest Academy, and medical students of the VU University attending their transition courses at Northwest Academy, attended four IPE-sessions over a period of one year. During each session, randomly paired couples of one medical student and one nursing student were asked to write a health care plan for a fictitious patient case that was discussed. The patient cases were constructed with the involvement of different experts, including clinical specialists, nurses, general practitioners, ensuring everyone’s perspective was well represented [21]. While discussing the patient’s problems and possible diagnostics or interventions, students learn with, from and about each other. In the periods in between these sessions, the students attended their scheduled internships at different hospitals.

In this study, ‘IPE’ refers to the four sessions in a classroom setting, where student pairs wrote health care plans together. ‘IPC’ refers to the interprofessional contacts students had during their internships in clinical practice.

### Participants

The nursing and medical students who followed this IPE-course were interviewed at the end of the year. The nursing students were third- and fourth-year students, following a four-year educational program. In the third and fourth year of their education, these students worked in clinical practice during 80% of the time and attended courses and training in the remaining 20%. The medical students were fifth year students, having finished a 3-year bachelor’s program and were halfway through their 3-year master’s program in medicine. They had finished four clerkships, including four transition courses.

Both groups of students had a wide range of experiences in clinical practice, and in different disciplines. Because of these experiences, we expected them to have had interprofessional contacts during different occasions and, therefore, to have a clear understanding of interprofessional collaboration in clinical practice.

The sampling was purposeful since participants were students that had experienced IPC after IPE-sessions and nursing and medical students were equally represented. We continued sampling until data sufficiency was reached (i.e. when no new information emerged from the interviews and the gathered data was sufficient to answer the research question) [22].

### Interviews

Semi-structured, face-to-face interviews were conducted using an interview guide with three main topics. To introduce the topic, each interview began with the question, ‘what’s your opinion about the classroom IPE-sessions that you attended?’. Subsequently students were asked about their experiences with and thoughts about interprofessional collaboration in clinical practice. The last question was, ‘what aspect from the classroom IPE-sessions do you take with you/will you remember when you participate in clinical practice?’.

### Procedure

All participants received an information letter about the research that explained its goals and procedures, and that participation in the interviews was voluntary. Immediately prior to the interviews, students were again informed about the duration, the anonymity and the audio recording of the interviews. All participants in this study signed an informed consent form. All interviews were conducted by the same person (CT) and the audio recordings were transcribed verbatim.

### Data analysis

All interviews were coded using ATLAS.ti. Thematic analysis was conducted, using open, axial and selective coding [23]. The open codes were established in three steps: firstly, four interviews were coded by two researchers of the team (CT and SB) independently, resulting an initial set of 17 codes, including the four sensitising concepts from social capital: ‘bonding’, ‘bridging’ and ‘linking’, and the ‘effect of social capital’. Secondly, all interviews were coded with this set of codes. A discussion followed about which topics required more detailed coding. Thirdly, all transcripts were coded again for the second and third interview questions with more detailed coding.

After the open coding process, the research team constructed the axial codes by discussing the main themes of the open codes. The selective coding was executed by comparing the axial codes, constructing a theory about their cohesion. All final themes and their cohesion were determined in discussion with the full research team.

### Ethical approval

The study was submitted to the local scientific board of Northwest Clinics, the Netherlands, which considers all study proposals within the institution. When a study falls within the scope of the Dutch Medical Research Involving Human Subjects Act (Sect. 1.b WMO, 26^th^ February 1998), a path of full approval is followed. Our study did not fall within this scope, so the committee waived their approval. All participants were informed that their participation was voluntary and that non-participation would not cause them harm.

### Reflexivity

The research team in this study consists of one sociologist, three medical doctors and two clinical specialists. The team was carefully assembled to balance out the different perspectives. All medical doctors were experienced in research in medical education and qualitative research methods and/or were closely involved in the guidance of medical and nursing students in clinical practice or in training setting. Having clinical specialists on the team helped to understand the experiences of students with IPC from the perspective of clinicians, while having a sociologist on the team aided in the interpretation the data from a perspective different from that of medical doctors.

One medical doctor who participated in this study (CT), conducted all interviews. She also taught the students included in this research. Being the teacher enabled interpreting the data and events the students described. Mentoring students during their internships for the last years, helped knowing students’ struggles and experiences in clinical practice, and helped to understand students’ language and the way they expressed themselves. To lower the chance of socially desirable answers, we informed the participants verbally and in the informed consent letter that the research and interviews were completely separate from, and had no influence on, the regular program. During the interviews, the interviewer also reassured and encouraged students, verbally and non-verbally, if they talked about negative opinions or experiences. In addition, all codes were discussed with another member of the team and the themes were finalized by the full research team.

## Results

Nine nursing students (7 female, 2 male) and thirteen medical students (6 female, 7 male) were interviewed up to 15 min per interview. Data sufficiency was reached after these 22 interviews.

### Influence of IPE on perceptions

According to the research question, the effect of IPE in classroom setting on students’ perceptions of IPC in clinical practice, was the focus of this study. As described in the methods section, students talked about what aspect from the IPE-sessions they will take with them or what they will remember, when they participate in clinical practice. These experienced effects of IPE were categorised into four themes: Exchange of discipline-specific knowledge, general knowledge about one’s own and the other one’s responsibilities, reduction of hierarchy, and improvement in patient care. These themes were related to each other. Their similarities and differences will be discussed in the following section. We will also discuss how each theme reflects an increase in social capital.

#### Exchange of discipline-specific knowledge

Some students learned specific knowledge from the other discipline, that they can apply in clinical practice. The ‘discipline-specific knowledge’ in this theme mainly concerns basic facts that are known by the other professional (e.g., the meaning of medical terms for nursing students). Sometimes it also concerns some background information about the clinical relevance of these basic facts, (e.g., why a specific diagnostic test is requested). This acquisition of discipline specific knowledge seems to appear primarily to nursing students.

*‘They have all explained the abbreviations of the laboratory diagnostics, in an understandable way’. (Student no.20-nursing)*

*‘For example, medication. Why and when you prescribe it to a patient. And why you will choose an alternative. That’s what I found interesting, how they make those choices’. (Student no.5-nursing)*

The acquisition of knowledge of the other profession is also an increase of social capital, while students have more access to (different) knowledge resources. This form of increased social capital reflects ‘bridging social capital’, since students experience that the other student is from a different group. In the quote below, a student points out that this acquisition and sharing of knowledge can also result in ‘getting ahead’ (i.e., ‘doing better’), which is also an important effect of increased social capital.

*‘That you benefit from each other, I mean you can come up with different things. That was informative. I think I wouldn’t have done as well, if I had done it on my own’. (Student no.4-medical)*

#### Understanding own and other’s responsibilities

Getting to know each other’s responsibilities was an important experience in the IPE-sessions for students. Several students stated that that experience was the most important effect of the IPE-sessions. This theme is connected to the first, while both represent learning from each other’s discipline-specific knowledge. However, the second theme involves more than the factual knowledge as described in the first theme. Students used the discipline-specific knowledge to construct a more general vision about their own and the other’s responsibilities.

*‘[It provided me] also insight, the medical students [we paired up with] were in their last year, I see such students also a lot in clinical practice, so now I know a little about what they do. And also with the doctor’s rounds, now you know what they do and what is our responsibility’. (Student no.21-nursing).*

This may resemble ‘understanding each other’s *clinical reasoning*’, but the knowledge they acquire in this theme concerns more (e.g., each other’s roles), and also a clarification of one’s own role.

*‘But I do think that because of the IPE-sessions, I realized more what my specific role as a clinician is’. (Student no.6-medical)*

#### Reduction of hierarchy

Both medical and nursing students argued for the reduction of hierarchy as an effect of the IPE-sessions. This theme is also connected to themes 2 and 4. Students pointed out that IPE lowered the barrier to communication by better understanding each other (Theme 2). Some point out the relevance of not feeling discouraged to talk, because that will harm the patients’ well-being (see Theme 4).

*‘I do think so [that IPE has influenced the way the student acts in clinical practice].. Because you have more intense contact with a medical student, with a doctor so to say. So… what I pointed out earlier about the difference between I am the doctor and you are the nurse, that has reduced’. (Student no.3-nursing).*

*‘I did see that occasionally, that nurses talk about doctors like ‘look how he does that, he really feels superior’, while that doctor maybe didn’t mean it that way at all. Or a doctor that feels ‘they don’t even know what that medication is’, but if you know that they are not educated with that knowledge, its reasonable that they don’t know. So I think understanding each other better is maybe a good thing’. (Student no.9-medical).*

*‘I believe this is an important thing to do, to just have a good time with each other. So the gap doesn’t become to big, if you understand what I mean. That one person is feeling superior and the other one is feeling anxious to talk. If they see you struggle as well, and they know you are also just learning, I think that is important.’ (Student no.9-medical).*

Students spoke often spontaneously about hierarchy in clinical practice, and how IPE can narrow this experienced gap. This reduction of hierarchy refers to the ‘linking’ or ‘scaling’ social capital, because students experience a difference in social status.

*‘I don’t know if it sounds a bit funny, but (I take with me) that they also would like to help you. Sometimes doctors have a higher… that maybe sounds a bit… but sometimes it really is like that. Especially with surgeons, they can really come across as intimidating. But actually, (…) they are just people our age. And they aren’t per se higher, they just want to help if you are struggling’. (Student no.19-nursing).*

#### Improvement in patient care

Some students highlighted the improvement in patients’ well-being as an effect of the IPE. The way patients benefit according to the students is by reducing the hierarchy (Theme 3) or professionals knowing each other’s responsibilities (Theme 2). The way this theme differs from the other two is that patients’ wellbeing is specifically pointed out, and the other factors are used to justify the statement.

*‘That the vision of us as a doctor, is not the only side of the story, and also not the best care for the patient per se. That there are more different points of care. We maybe order a blood test, additional medical examination, or antibiotics treatment, and I’ve noticed that nurses often think about bringing in a social worker or physical therapist, or how the patient will return home after this. So more broadly, seeing the picture of the patient. Also the social part’. (Student no.10-medical).*

This improvement in patient care is what is described in social capital theory as ‘getting ahead’: the care for patients is improving because of the benefits of enlarged social capital the students experience after IPE.

The four themes described above reflect bridging and linking social capital. Bonding social capital did not appear in our interviews.

### Barriers and facilitators to IPC

In addition to the influence of the IPE sessions, students also spontaneously talked about other factors that facilitated or hindered IPC in clinical practice. In the literature, these factors are often referred to as ‘barriers and facilitators’ to IPC [24, 25]. Some factors in this study were explained as both a barrier and facilitator, depending on the situation in which it occurred. All factors could be ordered into four themes: Perceived hierarchy, organisational factors, personal characteristics, and the feeling of responsibility for a patient.

Table 1 Depicts the four themes, all corresponding factors, if the factor was indicated as barrier or facilitator, and an exemplary quote for each factor.

**Table 1:** Experienced factors influencing IPC

Theme	Factor	Barrier (-) or facilitator ( +)	Quote
**Perceived hierarchy**	Hierarchy	-	*‘Surgeons are on a higher ranking compared to nurses […] that difference is still there. But on the internal medicine ward, with internists it is totally different. They are closer to the nursing staff, and they do actually listen to your opinion’. (Student no.5-nursing)*
**Organisational factors**	Time pressure	-	*‘You do notice immidiately if a doctor is very busy and doesn’t feel like explaining something’. (Student no.8-nursing)*
	Organisation of ward	-/ +	*‘If I’m on a ward, but I don’t have patients of my own, then I’ll be sitting on the side. But if I do participate with 3 or more patients… then yes, those are my patients and I’ll be addressed as an intern if a nurse is present. Then I feel part of the team’. (Student no.12-medical)*
	Being familiar with the ward	+	*‘I do feel that way now [feel comfortable in interprofessional contact], now that I have switched to a different ward. You first need to get used to a new ward. Because, at the other one, I felt so comfortable, I knew all ins and outs, that I enjoyed’. (Student no.5-nursing)*
**Personal**	Being a student	+	*‘They know you are a student, and I feel that we get to give more input because of that. Nurses [graduated] want to keep it short and move on. With us [students] they really want to explain things’. (Student no.11-nursing)*
		-	*‘I didn’t really have contact with nurses. Because what I said earlier, they usually turn to my supervisor, I’m not someone that can make something happen. If something needs to be taking care of for a patient, I can’t do that. So they go to the source directly’. (Student no.6-medical)*
	Age/level difference	-	*‘If I have to ask a surgeon something, that is of course different than a medical student […], he (student) is more the same age. Well, not the same age, but the difference is less. And they are also still learning, and we are too’. (Student no.22-nursing)*
	Character traits	-/ +	*‘Maybe it also helps that you are a little bit more assertive [when more experienced in education] and you dare to ask more questions’. (Student no.20-nursing)*
**Patient**	Responsibility for patient	+	*‘In clinical practice [the IPC] is for a real patient. And also, in clinical practice I feel like I’m the doctor, or at least: this is my responsibility that I need to take care of’ (Student no.6-medical)*

## Discussion

This qualitative study explored the influence of IPE-sessions in a classroom setting medical and nursing students’ perceptions of IPC in clinical practice. Social capital theory was used as the theoretical lens. This study reveals new insights in how IPE-sessions in classroom setting influences students’ perceptions of IPC in clinical practice, by increasing their social capital. These insights contribute to the understanding of how and why knowledge transfers between IPE in undergraduate curricula and IPC in clinical practice.

**Fig. 2:**
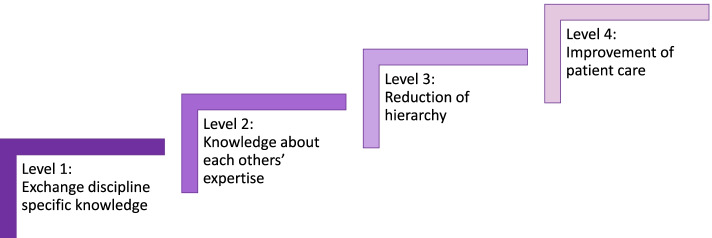
The four levels of learning effects of IPE found in this study

Most importantly, we studied the influence of IPE in a classroom setting on students’ perceptions of collaboration in clinical practice. Our results show four different, but related perceived influences of the IPE-sessions, which can be organised in steps. Each level builds on and is more complex than the previous one. Level 1 is the most easily achieved after IPE. On the other hand, Level 4 is less obvious for students to achieve, but it is a more desirable effect because students feel that patient care had actually improved. Figure 2 depicts how the different levels of effect, found in this study, are ordered.

All four levels in our study reflect an increase of social capital among students. Some students only learned specific items from the IPE-sessions (Level 1). Other students generalize the acquisition of this knowledge to more common knowledge about one’s own or others’ expertise (Level 2). The insight into the other’s expertise and the exchange of knowledge is commonly known to be a positive outcome of IPE [26–28]. Enlargement of these (knowledge) resources, as represented in Level 1 and 2, is described as one of the benefits of social capital. This refers to bridging social capital, since the knowledge or expertise belongs to a different group. Additionally, bridging social capital, IPE contributes to the enlargement of linking social capital among students as well. This is reflected by the reduction of hierarchy in Level 3. Apparently, students feel a difference in status, considering the way they describe the feeling of ‘hierarchy’. The way students think and feel about hierarchy in clinical practice – and thus linking social capital as a result of IPE – was a remarkable finding, since there were no questions about hierarchy in the interviews. Yet many students talked about it immediately when asked about their experiences with IPC. Finally, at Level 4, some students even experience that, if they know more about each other and/or there is less hierarchy, patient care will improve. Although several quotes of students at all four levels indicate that they experience some form of ‘getting ahead’, this last level is the most desirable form, because patient care actually improves.

Bonding social capital was the only form of social capital that did not appear in our interviews. This is coherent with the definition of ‘bonding social capital’: ties between individuals with that are close and strong, such as family or close friends. Furthermore, students in this study did not have a long-term relationship with each other, and thus strong and close ties could not be established. However, even though the relationships were not ongoing or longitudinal, students still experienced growth in bridging and linking social capital. This can occur because of the ‘capital’-nature of social capital: students can reinvest it in new interprofessional groups.

When evaluating the other barriers and facilitators to IPC of students in clinical practice, there were also similarities found with previous studies. Visser et al. (2017) [24] stated in a review study about barriers and facilitators to IPE that ‘feeling intimidated by doctors’ is a barrier, which is similar to the barrier ‘hierarchy’ in IPC. ‘Feelings of urgency because of a patient crisis’ is described as a facilitator by Visser et al. (2017) [24]. This seems similar to our facilitator ‘responsibility for patient’ in IPC, regarding the fact that a real patient in need – instead of a paper case description in classroom setting – stimulates students to take collaboration more seriously. The barriers and facilitators to IPC in this study can also be compared to the factors Olde Bekkink et al. (2018) [25] found for residents in the emergency department: personal, system, interpersonal and training. The ‘personal’ factor bears a resemblance to ‘personal’ in this study, while ‘system’ is similar to this study’s ‘organisational’ factor. ‘Interpersonal’ does have similarities to ‘perceived hierarchy’ in our study, since hierarchy was one of the interpersonal factors found in the study of Olde Bekkink et al. (2018) [25]. In our study, ‘perceived hierarchy’ was the only interpersonal factor that was found. The last factor ‘training’ in the study by Olde Bekkink et al. (2018) [25] obviously differs from our fourth factor of ‘the feeling of responsibility for a patient’. The similarity between the two factors is that students or residents described the two factors as being missed: for students there was no responsibility for a real patient in an educational setting, and for residents there was a lack of training in IPC. Students pointed these two factors out as, if present, facilitating better IPC.

**Fig. 3:**
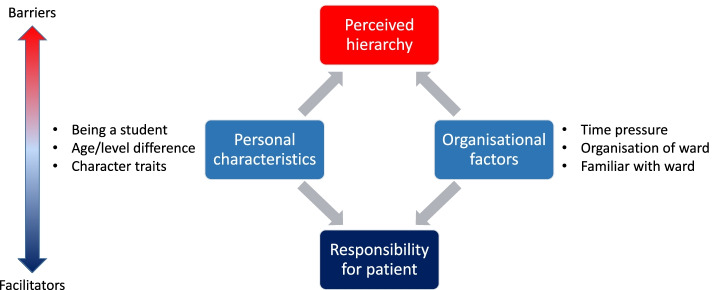
Barriers and facilitators and their interactions

In our research, in contrast with above studies, the factors were not only identified, but also the interaction between them could be constructed. In this study we found that personal and organisational factors could reduce or increase the perceived hierarchy or responsibility for the patient. Figure 3 depicts the interactions between the barriers and facilitators of this study.

For example, a difference in age (i.e., personal factor) can foster the feeling of hierarchy, but above all, some students are more sensitive about relationships than other students. Personal character traits are also of important influence of feeling responsible for the care of patients. Organisational factors can also influence the feeling of responsibility for patients (e.g., when students are more involved in the care for patients). Organisational factors can also influence the sense of hierarchy (e.g., ‘having a coffee break together’) can reduce the feeling of hierarchical differences. Visser et al. (2019) [9] also noticed that organisational factors encouraging informal contact, such as a shared room for different professionals, can stimulate the feeling of relatedness with the peers in the context.

This study is subject to some limitations. First, the results are based on one specific intervention at one specific educational facility. Therefore, results of this study, as in other qualitative studies, may have limited transferability to other interventions and facilities. However, the barriers and facilitators found in this study do seem to be similar to the barriers and facilitators found in other research. This may suggest that the perceptions we found in our study also show similarities if studied in different settings. Second, although students were explicitly reassured — in writing, verbally and non-verbally, before and during the interviews — that the research was independent of regular courses, students might have given socially desirable answers, considering their teacher was the interviewer. Third, in this setting longitudinal relationships between the students could not be established. This might have diminished the effect of growth in social capital. Fourth and last, the interviews were executed shortly after the last IPE-session. It is unclear if long term effects are similar. Further research on long-term effects is underway. Additional inquiry into the personal and educational factors that may influence deep learning effects of IPE-interventions is also recommended.

## Conclusion

This qualitative study explored which effect medical and nursing students experienced of IPE-sessions in a classroom setting on IPC in clinical practice. The results reveal effects on multiple levels, all reflecting an increase of social capital among students. In addition to indirect improvement in patient care because of, for example, enlargement of knowledge resources, students explicitly described that they experienced an improvement in patient care as a result of the IPE-sessions in classroom setting. These findings make classroom IPE-initiatives a valuable contributor to sustainable IPE, and justify educational facilities establishing and investigating in this form of IPE. Further research is necessary to determine when and why some students experienced deeper learning effects than others.

## Data Availability

The datasets used and analysed during the current study are available from the corresponding author upon reasonable request.
